# Multisystem Inflammatory Syndrome with Features of Atypical Kawasaki Disease during the COVID-19 Pandemic

**DOI:** 10.1155/2021/9950588

**Published:** 2021-10-08

**Authors:** Reza Shahriarirad, Anahita Sanaei Dashti, Shabnam Hajiani Ghotbabadi

**Affiliations:** ^1^Thoracic and Vascular Surgery Research Center, Shiraz University of Medical Science, Shiraz, Iran; ^2^Student Research Committee, Shiraz University of Medical Sciences, Shiraz, Iran; ^3^Professor Alborzi Clinical Microbiology Research Center, Nemazee Hospital, Shiraz University of Medical Sciences, Shiraz, Iran; ^4^Rheumatology Department, Shiraz Medical School, Shiraz University of Medical Sciences, Shiraz, Iran; ^5^Department of Allergy and Clinical Immunology, Shiraz University of Medical Sciences, Shiraz, Iran

## Abstract

**Background:**

Although Kawasaki disease (KD) is the most common self-limited systemic vasculitis in pediatrics, the exact etiology of the disease, its association with other diseases, and pathogens is still unknown. In order to achieve a better understanding and management of the disease, documentation and reporting of atypical cases is justified, particularly with the growing number of children with inflammatory syndrome with clinical features simulating KD during the COVID-19 pandemic. Here, we present a case of an atypical case of KD presenting as multisystem inflammatory syndrome (MIS) during the COVID-19 pandemic. *Case Presentation*. The patient is a 7-year-old girl who developed fever (39°C) and erythematous multiform rash on the abdomen and along with erythema and edema on the extremities. Laboratory evaluation revealed neutrophilia and lymphopenia along with elevated C-reactive protein, erythrocyte sedimentation rate, troponin, lactate dehydrogenase, ferritin, and D-dimer. Although the patient did not fulfill the KD criteria, based on approved guidelines and approaches regarding atypical KD and multisystem inflammatory syndrome in children (MIS-C) during the COVID-19 pandemic, intravenous immunoglobulin along with aspirin was administered for the patients. The patient's symptoms resolved with an uneventful postdischarge course.

**Conclusion:**

Early diagnosis and treatment of patients meeting full or partial criteria for KD are critical to preventing end-organ damage and other long-term complications, especially during times of public crisis and global health emergencies, such as the novel coronavirus pandemic.

## 1. Introduction

Kawasaki disease (KD) is the most common and self-limited systemic vasculitis in pediatrics, mainly affecting the small and medium-sized vessels [1]. Despite several decades of investigation, the etiology of KD remains unknown and the full spectrum of disease is not yet understood; therefore, in order for the better understanding of the disease and achieving satisfactory therapeutic results, characterization of this syndrome and its risk factors, along with the gathering of standardized data demonstrating the clinical presentations, severity, epidemiology, and outcomes of the disease is justified. Here, we present a 7-year-old girl who presented with atypical features of KD and multiorgan dysfunction in April 2020, during the COVID-19 pandemic.

## 2. Case Presentation

The patient is a 7-year-old girl, with no significant medical history and recent history of presence in populated areas and social gathering, who developed small lesions with pruritis on her lower extremities, which extended during the next two days and further on, developed fever (38.5°C). She visited a family physician and was prescribed with antihistamine drugs and acetaminophen, which showed no improvement. Therefore, she visited a pediatric specialist on fever and lesion, especially on the hands, legs, and groin, and was admitted to a local hospital with a provisional impression of Kawasaki disease. Due to no improvement in the condition of the patient and a need for echocardiography by pediatric cardiologist, she was transferred to our center, as a referral and specialty pediatric hospital, for further evaluation.

**Figure 1:**
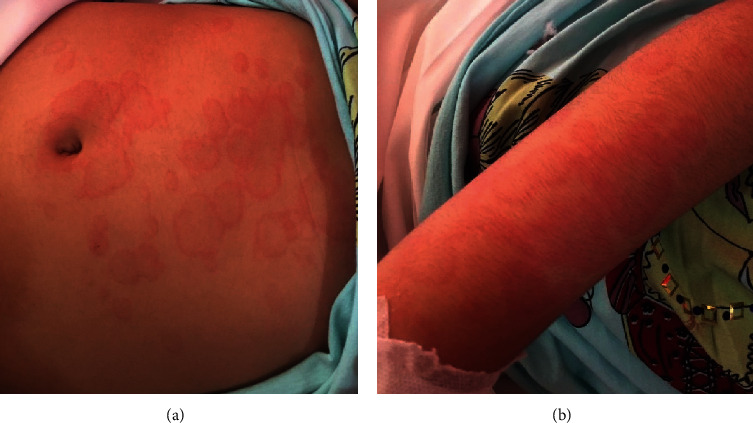
Erythematous multiform rash on the (a) abdomen and (b) upper extremity of a 7-year-old girl with atypical Kawasaki disease.

In our primary evaluation, the patient had now, after 6 days of the initiation of the symptoms, stable vital signs, only a temperature of 39°C, fully oriented (GCS:15), and erythematous multiform rash on the abdomen and upper and lower extremities (Figure 1), along with joint pain, subcutaneous edema, and erythema of the dorsal side of the hand without obvious tenderness. Based on the achieved history from the patient and her parents, the abdominal rashes were the last place of involvement. She also reported a history of conjunctivitis which was resolved at the time of admission in our center. The patient also developed scaling of the perianal area, which then progressed to scaling of the fingertips and toes after discharge. Laboratory evaluation of the patient is given in Table 1, which were in favor of the inflammatory process. Other laboratory data included elevated troponin levels, albumin, and normal antistreptolysin O (ASO) titer, aspartate transaminase (AST), alanine aminotransferase (ALT), negative blood culture, and cold agglutination test and also a normal urine analysis test. Abdominal pelvic sonography was also unremarkable.

Echocardiography was done for the patient who revealed good left and right ventricle systolic function with trivial pulmonary and tricuspid valve regurgitation and also a slight brightening of the right coronary artery without dilation, aneurysm, or clot (right coronary artery: 2.1 mm, left coronary artery: 1.9 mm, and left anterior descending artery: 1.8 mm).

Since the time of admission was during the COVID-19 pandemic, polymerase chain reaction (PCR) of COVID-19 and chest radiographs were requested, in which PCR was negative and a spiral chest computed tomography (CT) scan with IV contrast demonstrated subsegmental collapse in right and also in base of the left lung which were not typical for COVID-19.

Based on the mentioned findings, which were fever for more than 5 days, the erythematous multiform rash of the hands and legs, conjunctivitis, high ESR, and CRP level, the patient did not fulfill the criteria for complete KD and also incomplete KD based on the AHA algorithm including additional laboratory markers; However, based on the WHO definition for suspected multisystem inflammatory syndrome (MIS) in children and adolescents related to COVID-19 [2], intravenous immunoglobulin (IVIG) with a dose of 2 g/kg along with aspirin (80 mg per day, oral) was administered for the patient. The patients' fever relieved after receiving IVIG, and the rashes started to fade after 2 days of receiving IVIG, starting with the first locations of involvement, which was the lower and upper extremities, followed by the abdomen. Regarding the laboratory data of the patient, CRP levels decrease from above 150 mg/L, 80, and then to 4.8 and WBC levels from 7.8 to 4.5 (×10^9^/L); however, ESR levels increase from 66 to 100 mm/h.

The patient was discharged after 5 days of hospitalization with an uneventful postop course, and only mild scaling of the hands and feet continued. The follow-up laboratory test demonstrated a positive value of immunoglobulin G (IgG) antibody for SARS-CoV-2.

## 3. Discussion and Conclusion

KD is an acute vasculitis of childhood, with 50% of cases occurring in those under two years of age and 80% in those under five years of age, and is the leading cause of acquired heart disease in pediatrics in developed countries [3]. Despite that KD affects children from all races, it is most frequent in Asian populations. Our case presents an atypical case of KD which did not fill the full criteria and was not at the typical patient's age for the disease. Taking into consideration these presentations and features is advised especially during the times of public crisis and emergencies.

In this regard, COVID-19 which was declared a pandemic and global emergency by the WHO on 11th March 2020 has seized the attention of medical staff, governments, and people and affected other disease management and diagnosis approaches. Among the group at risk are children, with the disease affecting them in various ways [4–6]. There is a growing global concern that SARS-CoV-2-related inflammatory syndrome is emerging in children. Multiple infectious triggers such as adenovirus and New Haven coronavirus have been inconclusively associated with KD in the past [7, 8]. Also, KD has been in associations with Epstein–Barr virus (EBV), rotavirus, and bacteria [9, 10]. In this regard, the WHO has also developed a preliminary case definition and case report form for MIS in children and adolescents for approaching these diseases [2].

Lately, reports from North America and Europe have described clusters of children and adolescents requiring admission to intensive care units due to MIS accompanied by some characteristics similar to those of KD and toxic shock syndrome. Case reports and small series have described the presentation of acute illness accompanied by a hyperinflammatory syndrome, leading to multiorgan failure and shock [11–13]. Laboratory features of these patients included neutrophilia, high CRP levels which were also similar in our case and other case reports [14, 15]. Furthermore, in our case, the patient was located in a COVID-19 hotspot area [16, 17] with a recent history of attending public places. Hence, it could have been possible that she developed a previous asymptomatic infection, as the positive IgG for SARS-CoV-2 in our patient is in favor of this theory.

Asymptomatic COVID-19 in pediatric is not an uncommon feature, while some patients may also report late onset and even atypical symptoms, such as skin manifestations and loss of appetite. Therefore, the correct interpretation of gustatory and skin symptoms associated with specific laboratory tests for SARS-CoV-2 infection can lead to the most appropriate management and supportive care, due to the unique immune characteristics of pediatric patients [18, 19]. In summary, timely diagnosis of COVID-19 is still a challenging issue which requires further reports from centers and expert clinicians worldwide, until efficient management and control is applied [20–22].

A leading hypothesis is that MIS may be related to COVID-19 based on initial laboratory testing showing positive serology in a majority of patients. Children have been treated with anti-inflammatory treatment, including parenteral immunoglobulin and steroids.

The relationship between COVID-19 and KD is still unknown; however, a possible explanation may be in PCR-negative antibody-positive cases; previous infection by the coronavirus could possibly trigger an immune response simulating KD. Furthermore, COVID-19 during the active infective phase has been linked to excessive cytokine and inflammation storm similar to KD.

Similar reports from the USA regarding MIS in pediatrics with features similar to our cases such as rash, high CRP and troponin levels, and negative PCR for COVID-19 have been published [13, 22, 23]. Based on our knowledge, this is the first report regarding MIS simulating atypical KD during the COVID-19 pandemic in Iran. Rauf et al. also reported a similar case of MIS presenting as atypical KD in India with negative PCR for COVID-19 [15]. However, unlike our case, the patient in their study presented with myocarditis.

Based on similar reports from numerous countries with temporal relation to COVID-19 infection in the community, it is essential for general pediatricians to be on alert for such atypical presentations, and early referral to tertiary care should be considered as appropriate. Furthermore, if MIS is suspected, pediatricians should immediately refer patients to a specialist in pediatric infectious disease, rheumatology, and/or critical care, as indicated. Early diagnosis and treatment of patients meeting full or partial criteria for KD is critical to preventing end-organ damage and other long-term complications.

## Figures and Tables

**Table 1:** Laboratory information on admission of a 7-year-old girl in favor of multisystem inflammatory syndrome with features of atypical Kawasaki disease.

Test	Value	Normal reference range
C-reactive protein	3+	Negative
Erythrocyte sedimentation rate (mm/h)	66	0–30
Ferritin (ng/mL)	130	4.6–204.0
Platelets (×10^3^/*µ*L)	169	150–450 × 10^3^/mL
White blood cell count (×10^3^/*μ*L)	7.8 × 10^9^	4.5–14.5 × 10^3^/*µ*L
Lymphocyte	1.56	1.5–7.0 × 10^3^/*µ*L
Hemoglobin (g/dl)	11.3	11.5–15.5
Lactate dehydrogenase (U/L)	518	60–170
Procalcitonin (ng/ml)	0.47	0.10–0.49
D-dimer (mg/ml)	2.20	0.4–2.27
Fibrinogen (mg/dL)	357	200–400

## Data Availability

The data used to support this study are included within the article and are available from the corresponding author upon request.

## Abbreviations

ALT: Alanine aminotransferase
ASO: Antistreptolysin O (ASO) titer
AST: Aspartate transaminase
COVID-19: Coronavirus disease 2019
CRP: C-reactive protein
CT: Computed tomography
EBV: Epstein–Barr virus
ESR: Erythrocyte sedimentation rate
GCS: Glasgow Coma Scale
IgG: Immunoglobulin G
IVIG: Intravenous immunoglobulin
KD: Kawasaki disease
LDH: Lactate dehydrogenase
MIS: Multisystem inflammatory syndrome
MIS-C: Multisystem inflammatory syndrome in children
PCR: Polymerase chain reaction
SARS-CoV-2: Severe acute respiratory syndrome coronavirus 2
WHO: World Health Organization.

## References

[1] Levy M., Koren G. (1990). Atypical Kawasaki disease. *The Pediatric Infectious Disease Journal*.

[2] World Health Organization (2020). *Multisystem Inflammatory Syndrome in Children and Adolescents with COVID-19: Scientific Brief, 15*.

[3] Rowley A. H., Shulman S. T. (2018). The epidemiology and pathogenesis of Kawasaki disease. *Frontiers in Pediatrics*.

[4] Shahriarirad R., Erfani A., Ranjbar K., Bazrafshan A., Mirahmadizadeh A. (2021). The mental health impact of COVID-19 outbreak: a Nationwide Survey in Iran. *International Journal of Mental Health Systems*.

[5] Mirahmadizadeh A., Ranjbar K., Shahriarirad R. (2020). Evaluation of students’ attitude and emotions towards the sudden closure of schools during the COVID-19 pandemic: a cross-sectional study. *BMC Psychology*.

[6] Ranjbar K., Hosseinpour H., Shahriarirad R., Ghaem H., Mirahmadizadeh A., Hosseinpou P. (2021). Students’ attitude and sleep pattern during school closure following COVID-19 pandemic quarantine: a web-based survey in south of Iran. *Environmental Health and Preventive Medicine*.

[7] Jaggi P., Kajon A. E., Mejias A., Ramilo O., Leber A. (2013). Human adenovirus infection in Kawasaki disease: a confounding bystander?. *Clinical Infectious Diseases*.

[8] Esper F., Shapiro E. D., Weibel C., Ferguson D., Landry M. L., Kahn J. S. (2005). Association between a novel human coronavirus and Kawasaki disease. *The Journal of Infectious Diseases*.

[9] Maggio M. C., Fabiano C., Corsello G. (2019). Kawasaki disease triggered by EBV virus in a child with Familial Mediterranean Fever. *Italian Journal of Pediatrics*.

[10] Son M. B., Sundel R. P., Petty R. E., Laxer R. M., Lindsey C. B., Wedderburn L. R. (2016). Kawasaki disease. *Textbook of pediatric rheumatology*.

[11] Riphagen S., Gomez X., Gonzalez-Martinez C., Wilkinson N., Theocharis P. (2020). Hyperinflammatory shock in children during COVID-19 pandemic. *The Lancet*.

[12] DeBiasi R. L., Song X., Delaney M. (2020). Severe Coronavirus disease-2019 in children and young adults in the Washington, DC metropolitan region. *Jornal de Pediatria*.

[13] Jones V. G., Mills M., Suarez D. (2020). COVID-19 and Kawasaki disease: novel virus and novel case. *Hospital Pediatrics*.

[14] Royal College of Paediatrics and Child Health (2020). Guidance- COVID-19 paediatric multisystem inflammatory syndrome. https://www.rcpch.ac.uk/resources/guidance-paediatricmultisystem-inflammatory-syndrome-temporally-associatedcovid-19.

[15] Rauf A., Vijayan A., John S. T., Krishnan R., Latheef A. (2020). Multisystem inflammatory syndrome with features of atypical Kawasaki disease during COVID-19 pandemic. *Indian Journal of Pediatrics*.

[16] Shahriarirad R., Khodamoradi Z., Erfani A. (2020). Epidemiological and clinical features of 2019 novel coronavirus diseases (COVID-19) in the South of Iran. *BMC Infectious Diseases*.

[17] Sabetian G., Moghadami M., Hashemizadeh Fard Haghighi L. (2021). COVID-19 infection among healthcare workers: a cross-sectional study in southwest Iran. *Virology Journal*.

[18] Maniaci A., Iannella G., Vicini C. (2020). A case of COVID-19 with late-onset rash and transient loss of taste and smell in a 15-year-old boy. *American Journal of Case Reports*.

[19] Guarneri C., Rullo E. V., Pavone P. (2021). Silent COVID-19: what your skin can reveal. *The Lancet Infectious Diseases*.

[20] Shahriarirad R., Fallahi M. J. (2020). TB and the COVID-19 pandemic: brothers in arms against lung health. *International Journal of Tuberculosis & Lung Disease*.

[21] Shahriarirad R., Sarkari B. (2021). COVID-19: clinical or laboratory diagnosis? A matter of debate. *Tropical Doctor*.

[22] New York City Department of Health and Mental Hygiene Pediatric multi-system inflammatory syndrome potentially associated with COVID. http://www1.nyc.gov/assets/doh/downloads/pdf/han/alert/2020/covid-19-pediatric-multi-system-inflammatory-syndrome.pdf?referringSource=articleShare.

[23] Dashti A. S., Ebrahimi K., Shahriarirad R., Ghotbabadi S. H., Aminnia S. (2021). COVID-19 pandemic in the disguise of multi system inflammatory syndrome in children: a case series. *Research Square*.

